# Supplementary material for: Analysis of Trypanosoma equiperdum Recombinant Proteins for the Serological Diagnosis of Dourine
Source: Vet Sci. 2024 Mar 13;11(3):127. doi: 10.3390/vetsci11030127 (PMC10974970; doi:10.3390/vetsci11030127)

Figure S1. SDS-PAGE and Coomassie stain of purified proteins A0A1G4I8N3, A0A1G4I464 and A0A1G4I740. Lane M: molecular weight standard (Novex Sharp Prestained Protein Standard, Life Technologies); lane 1: protein A0A1G4I8N3; lane 2: protein A0A1G4I464; lane 3: protein A0A1G4I740.

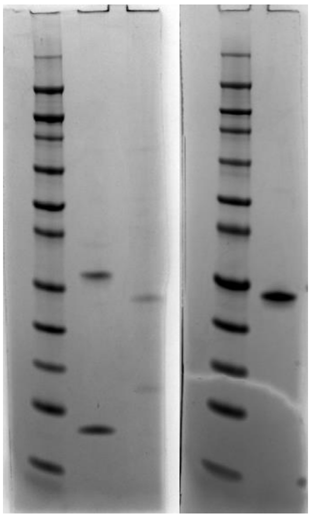

Figure S2. (a) Western blotting test using *T. equiperdum* OVI whole antigen (lane 1) and recombi-nant proteins A0A1G4I8N3 (lane 2), A0A1G4I464 (lane 3) and A0A1G4I740 (lane 4) incubated with the reference horse serum positive for T. equiperdum (batch 019/1994, IZSAM); **(b)** *T. equiperdum* OVI whole antigen (lane 1) and recombinant proteins A0A1G4I8N3 (lane 2), A0A1G4I464 (lane 3) and A0A1G4I740 (lane 4) incubated with the reference horse serum negative for *T. equiperdum*. Lane M: molecular weight standard (Novex Sharp Prestained Protein Standard, Life Technologies).

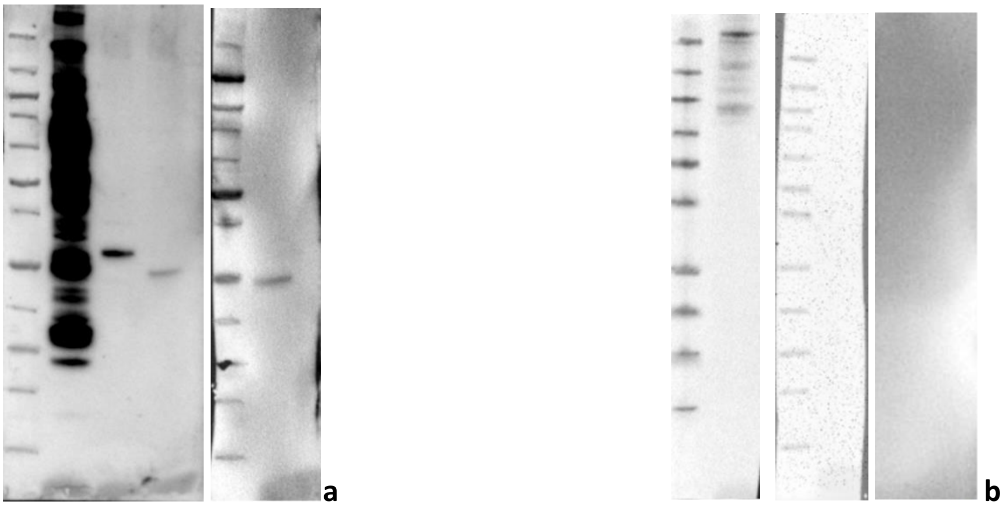

Supplement: Supplementary file 1 [file vetsci-11-00127-s001.zip › vetsci-2862213-supplementary.pdf]
